# Effect of a Website That Presents Patients’ Experiences on Self-Efficacy and Patient Competence of Colorectal Cancer Patients: Web-Based Randomized Controlled Trial

**DOI:** 10.2196/jmir.7639

**Published:** 2017-10-13

**Authors:** Jürgen M Giesler, Bettina Keller, Tim Repke, Rainer Leonhart, Joachim Weis, Rebecca Muckelbauer, Nina Rieckmann, Jacqueline Müller-Nordhorn, Gabriele Lucius-Hoene, Christine Holmberg

**Affiliations:** ^1^ Section of Health Services Research and Rehabilitation Research, Medical Center – University of Freiburg, Faculty of Medicine, University of Freiburg Freiburg Germany; ^2^ Institute of Public Health Charité - Universitätsmedizin Berlin, corporate member of Freie Universität Berlin, Humboldt-Universität zu Berlin, and Berlin Institute of Health Berlin Germany; ^3^ Hasso-Plattner-Institute Potsdam Germany; ^4^ Department of Psychology University of Freiburg Freiburg Germany; ^5^ Clinic for Oncological Rehabilitation, UKF Reha Department of Psycho-Oncology University Clinic Center Freiburg Germany

**Keywords:** self-efficacy, colorectal cancer, patient competence, narrative information, Web-based experiential information

## Abstract

**Background:**

Patients often seek other patients’ experiences with the disease. The Internet provides a wide range of opportunities to share and learn about other people’s health and illness experiences via blogs or patient-initiated online discussion groups. There also exists a range of medical information devices that include experiential patient information. However, there are serious concerns about the use of such experiential information because narratives of others may be powerful and pervasive tools that may hinder informed decision making. The international research network DIPEx (Database of Individual Patients’ Experiences) aims to provide scientifically based online information on people’s experiences with health and illness to fulfill patients’ needs for experiential information, while ensuring that the presented information includes a wide variety of possible experiences.

**Objective:**

The aim is to evaluate the colorectal cancer module of the German DIPEx website krankheitserfahrungen.de with regard to self-efficacy for coping with cancer and patient competence.

**Methods:**

In 2015, a Web-based randomized controlled trial was conducted using a two-group between-subjects design and repeated measures. The study sample consisted of individuals who had been diagnosed with colorectal cancer within the past 3 years or who had metastasis or recurrent disease. Outcome measures included self-efficacy for coping with cancer and patient competence. Participants were randomly assigned to either an intervention group that had immediate access to the colorectal cancer module for 2 weeks or to a waiting list control group. Outcome criteria were measured at baseline before randomization and at 2 weeks and 6 weeks

**Results:**

The study randomized 212 persons. On average, participants were 54 (SD 11.1) years old, 58.8% (124/211) were female, and 73.6% (156/212) had read or heard stories of other patients online before entering the study, thus excluding any influence of the colorectal cancer module on krankheitserfahrungen.de. No intervention effects were found at 2 and 6 weeks after baseline.

**Conclusions:**

The results of this study do not support the hypothesis that the website studied may increase self-efficacy for coping with cancer or patient competencies such as self-regulation or managing emotional distress. Possible explanations may involve characteristics of the website itself, its use by participants, or methodological reasons. Future studies aimed at evaluating potential effects of websites providing patient experiences on the basis of methodological principles such as those of DIPEx might profit from extending the range of outcome measures, from including additional measures of website usage behavior and users’ motivation, and from expanding concepts, such as patient competency to include items that more directly reflect patients’ perceived effects of using such a website.

**Trial Registration:**

Clinicaltrials.gov NCT02157454; https://clinicaltrials.gov/ct2/show/NCT02157454 (Archived by WebCite at http://www.webcitation.org/6syrvwXxi)

## Introduction

Activating patients to become partners in their care has been a priority in health policy in many Western countries over the past years [1]. Increasing patient participation in health care rests, however, on at least three interrelated prerequisites. First, information on disease, treatments, and outcomes should be widely available. Second, health care providers should be able to effectively convey this information to patients and enable them to make informed decisions. Third, patients should be able to access, process, decide, and act on the health information relevant to them. As a consequence, interest has grown in concepts that describe patients’ abilities to acquire and process health information, such as empowerment [2-5], health literacy [6-9], patient competence [10], and self-efficacy in coping with cancer [11-13] and other chronic diseases [14,15]. Obviously, the importance of these concepts can be rated even more highly if one considers the opportunities that the Internet provides for disseminating health information.

Although medical information on diseases, treatments, and outcomes that is based on available quality criteria [16-19] represents an important input into patients’ informed (treatment) decision making, patients facing health care decisions also seek and use experiential information describing how other patients live with a disease [20-22]. This field of experiential knowledge and its presentation has recently received increasing attention in research on health information resources. Here again, the Internet provides a wide range of opportunities to share and learn about other people’s health and illness experiences via blogs or patient-initiated online discussion groups, which may provide support from peers [5,23]. Some peer-to-peer online tools have been shown to increase patient empowerment in relation to information, mental health, and feeling supported [24,25], whereas others have revealed mixed or negative effects [5]. In addition, there exists a range of medical information devices that include experiential patient information, including decision aids [26] and other health information venues [27,28]. However, there is serious concern about the use of narratives in health information because they are powerful and persuasive tools and they may unduly influence health care decision making [29-33].

Against this background, this study asks whether a website that provides experiential information on living with colorectal cancer based on scientifically rigorous data collection and analysis positively influences self-efficacy for coping with cancer [11-15] and patient competence [10]. It aims to evaluate potential effects of a specifically designed website, while at the same time providing insight into factors contributing to changes in coping self-efficacy and patient competence that are increasingly attracting more research interest in psycho-oncology [10,34]. More specifically, we hypothesized that, compared to a waiting list control group, having access to and using a website presenting a broad range of individual experiences with colorectal cancer would increase patients’ perceived self-efficacy for coping with cancer and patient competencies such as the ability to manage emotional distress arising in the context of cancer and its treatment or self-regulation as an ability to maintain a satisfactory equilibrium in interactions and affect [10].

## Methods

### Study Design

The study used a randomized two-group between-subjects design with repeated measures; participants were randomly assigned either to an intervention group that had immediate access to the colorectal cancer module for 2 weeks or to a waiting list control group that was given access to the module after completion of the study 6 weeks after randomization. Coping self-efficacy served as the primary outcome; patient competencies constituted the secondary outcome. Outcome criteria were measured in both groups at baseline before randomization and at 2 weeks (time 1). At 6 weeks after baseline (time 2), follow-up measures were taken to test for the short-term stability of the intervention. The trial was conducted before the website became available to the general public.

The study protocol was approved by the Charité Universitätsmedizin Berlin ethics committee (EA4/053/12) and was registered (clinicaltrials.gov NCT02157454). This trial is reported according to the Consolidated Standards of Reporting Trials (CONSORT) statement and the CONSORT-EHEALTH extension.

### Intervention

The intervention of the study consisted of a website providing information of experiences by men and women diagnosed with colorectal cancer. The website is a section (module) of a German website krankheitserfahrungen.de, which aims to provide scientifically collected and analyzed experiences of health and illness to patients, health care providers, and the wider public. The project team DIPEx Germany that runs the website is a member of the international research network DIPEx (Database of Individual Patients’ Experiences) [35]. The website is hosted at the University of Freiburg. DIPEx aims to present online information on people’s experiences with health and illness that have been systematically collected through qualitative interviews and analyzed with rigorous qualitative research methods. DIPEx intends to fulfill patients’ needs for experiential information, while ensuring that the presented information includes a wide variety of possible experiences with the disease [36]. This is achieved by collecting illness experiences employing a maximum variation sampling strategy and using narrative interviewing techniques [37]. A researcher handbook details how the experiences are to be collected and analyzed. The handbook also ensures that funding for modules may only be provided by organizations with no involvement or financial interests in the content. This systematic and scientific approach and the transparency regarding funding distinguishes DIPEx markedly from peer-to-peer and other online support resources.

The content of the modules of the DIPEx website can be accessed via thematic pages (eg, “stoma” or “living with colorectal cancer”) or by interviewed persons (person pages). The person pages can be searched applying a filter (eg, age or gender) for ease of navigation. These features of the website are positively viewed by users [37-39]. In particular, the feature that one may find others who are similar to oneself seems to help users find hope [37].

### Sample Size and Power Calculation

In determining the necessary sample size for the trial, it was assumed that given a standard deviation of 18 [40,41], a difference of five scale points in self-efficacy for coping with cancer between the intervention group and the waiting list control group could be reasonably expected and should be detected with a power of .80 and a type one error probability of alpha=.05. For Cohen's *d* (between-group mean difference divided by SD) [42], this lies in the range of a small effect size (*d*=.20-.49). Based on these premises, a sample size of n=205 participants per group was deemed necessary.

### Eligibility Criteria

Potential participants were considered eligible if they were German-speaking, 18 years of age or older, and had either been diagnosed with colorectal cancer within the past 3 years before enrollment or—independent of time since diagnosis—had metastasized colorectal cancer and/or a relapse of the disease, and who consented to participate online on the study website. Potential participants who indicated on the survey that the time since their diagnosis was more than 3 years and who indicated that they had no recurrence or metastases were excluded from the study.

### Recruitment and Enrollment

A wide range of recruitment strategies was used. Many major websites related to colorectal cancer, such as felix-burda-stiftung.de and lebensblicke.de, were informed about the study and provided a link to the study website. Information on the study was posted regularly on a colorectal cancer Facebook group and on online colorectal cancer discussion groups. The study was presented in-person to support groups, in rehabilitation clinics, and to hospital staff for them to aid in recruitment. Some colorectal cancer centers also informed their patients about the study. Finally, the project was presented at information events for patients at hospitals and cancer meetings. Recruitment started in June 2014 and ended in August 2015.

Study participants had to enroll themselves through the study website, which also provided detailed information about the study. If participants then chose to enroll, this was considered as giving consent because they had previously been informed. After enrollment, participants were first asked to complete the baseline measures and were then randomized.

### Data Collection

Online data collection at the three measurement points required patients to complete validated questionnaire measures of self-efficacy for coping with cancer, patient competence, depression, social support, health-related quality of life, and medical information received (Table 1). In addition, information on selected sociodemographic, illness, and treatment characteristics was obtained at baseline. Participants were also asked to provide information on their use of Internet resources addressing issues related to colorectal cancer. The pages that each participant visited on the intervention website were logged along with a time stamp, thus allowing a determination of the amount of time they spent on the website (in minutes), the number of sessions using the site, and the number of clicks produced (as an indicator of the number of subpages accessed). The analyses reported here focus primarily on the results regarding self-efficacy for coping with cancer and patient competence.

### Primary Outcome: Self-Efficacy for Coping With Cancer

Self-efficacy for coping with cancer may be defined as a patient’s confidence in his or her ability to perform coping behaviors in the context of cancer [13]. As the primary outcome of this study, self-efficacy for coping with cancer was measured using the German version of the brief form of the Cancer Behavior Inventory (CBI-B-D) [40,43]. Like the CBI-B, the original version of this instrument [11,13], the CBI-B-D consists of 14 items that describe coping behaviors in the context of cancer. Patients are asked to rate how confident they are in performing each of these behaviors on a nine-point scale ranging from “not at all confident” to “totally confident.” A summary score is obtained across all 14 items, which can range from 14 to 126, with high values indicating high confidence in one’s ability to perform the coping behaviors. The German version was created using a forward-backward translation approach. Reliability estimates for both the original and the German versions of the scale are generally high. Furthermore, the validity of the scale has been demonstrated in various studies of concurrent, predictive, or construct validity [11,13]. The CBI-B-D score was measured at baseline and at 2 and 6 weeks postbaseline.

**Table 1:** Data collection: measurements and time points.

Variables measured^a^	Baseline	2 weeks into study (postintervention)	6 weeks after randomization
Sociodemographics; illness and treatment characteristics; Internet use behavior	X		
Depression: PHQ-2	X		
Social support: SSUK-8	X		
Self-efficacy for coping with cancer: CBI-B-D (primary outcome)	X	X	X
Self-ratings of patient competencies: FEPK 2-57 (secondary outcome)	All 8 subscales	4 subscales	4 subscales
Information: EORTC QLQ-INFO25		X	
Ratings of personal reports of those affected by cancer		X	X
Quality of life: EORTC QLQ-C30		X	

^a^CBI-B-D: German version of brief form of Cancer Behavior Inventory; EORTC QLQ-C30: questionnaire to assess the quality of life of cancer patients by the European Organisation for Research and Treatment of Cancer; EORTC QLQ-INFO25: questionnaire to assess information given to cancer patients; FEPK 2-57: 57-item questionnaire on patient competence using five problem-focused and three emotion-focused subscales; PHQ-2: two-item Patient Health Questionnaire; SSUK-8: German brief version of the illness-specific Social Support Scale.

### Secondary Outcome: Patient Competence

Following Giesler and Weis [10], patient competence in the context of cancer may be understood as a patient’s ability to deal with the tasks and distress arising from cancer and its treatment, to be guided by his or her personal needs and goals, and to make use of support available from significant others or from the health care system as a whole. Based on this working definition of patient competence, as well as factor analysis, they constructed a self-rating measure of patient competence (FEPK 2-57) that assesses five problem-focused and three emotion-focused competencies. The measure contains 57 items each rated on a five-point scale intended to measure behaviors indicative of patient competence as determined in prior pilot studies. Items addressing emotion-focused competencies offer the additional response option of “not applicable to me.” Subscale internal consistencies (Cronbach alpha) range from .64 to .87 (median .77) and may be judged as at least satisfactory. In this study, all these competencies were measured at baseline. At weeks 2 and 6, however, only the three emotion-focused competencies and one of the problem-focused competencies were measured as secondary outcomes because they were considered to best reflect the potential effects of the website.

The competencies measured at baseline and weeks 2 and 6 were “self-regulation” (ability to negotiate needed support and to allow for resting periods during the course of the day when needed), “managing distressing emotions” (ability to deal with cancer-related fears), “dealing explicitly with the threat posed to life by cancer” (being able to confront the idea that one might die), and “(low) avoidance” (ability not to engage in ruminating thoughts and avoidance behaviors) [10]. Problem-focused competencies measured only at baseline were “seeking information concerning disease and treatment,” “being assertive in interactions with physicians,” “striving for autonomous decisions,” and “interest in social services.” Scale scores for all competencies were formed by computing a participant’s individual mean across the respective items. Scores can vary between 1 and 5, with higher scores indicating a higher level of self-rated competence.

### Additional Measures

To allow a more comprehensive characterization of the participants, depression, social support, quality of life, and satisfaction with information received on the condition and its treatment were measured. Depression was measured at baseline using the two-item Patient Health Questionnaire (PHQ-2) [44,45]. Also at baseline, social support was measured by means of the SSUK-8 [46], the brief form of the German adaptation of the illness-specific Social Support Scale (SSUK) [47]. At 2 weeks, health-related quality of life was measured with the QLQ-C30, a reliable and valid instrument developed by the European Organisation for Research and Treatment of Cancer (EORTC) [48]. Finally, respondents’ evaluation of disease and treatment information received was measured with the EORTC QLQ-INFO25 [49] at 2 weeks.

### Data Analysis

Data analysis was performed using IBM SPSS versions 23 and 24. Baseline differences between the intervention and the waiting list group were analyzed by means of chi-square statistics for categorical variables or one-way analyses of variance (ANOVAs) in the case of continuous variables based on all participants with nonmissing data for a given variable. Effect sizes were estimated by computing phi coefficients or eta squared. Following Cohen [42], these may be categorized as small, medium, or large, with values of .10, .30, and .50 representing corresponding effect size thresholds for the phi coefficient, and values of .01, .06, and .14 representing those for eta squared.

For testing the hypothesized intervention effect on the primary and secondary outcomes, we preformed separate regression analyses of the postintervention (week 2) and follow-up (week 6) scores with the intervention dummy coded (intervention=0, control=1) and the respective pretest scores as an additional predictor, which is equivalent to a traditional analysis of covariance. These analyses were based on all randomized participants, using multiple imputation of missing values at baseline and at 2 and 6 weeks. We ran 10 multiple imputations with the full information maximum likelihood method when data were missing in single items or scales. Multiple imputation creates multiple datasets, in which the missing observations are imputed, using a stochastic algorithm that estimates values based on given information and creates different imputed values in each dataset. Statistics are performed separately for these datasets and coefficients are combined after having finished the analyses [50]. The assumption that data were missing completely at random could be retained after performing Little’s missing completely at random test [51], which was not significant with χ^2^_31,997_=27,222.8 (*P*>.99). The effect sizes of the predictors in the regression models are reported as beta weights for which the minima and maxima across the analyzed imputed datasets will be given. Following Cohen [42], values of .10, .30, and .50 for beta represent the thresholds for interpreting effects as small, medium, or large, respectively.

## Results

### Sample Characteristics at Baseline

**Figure 1:**
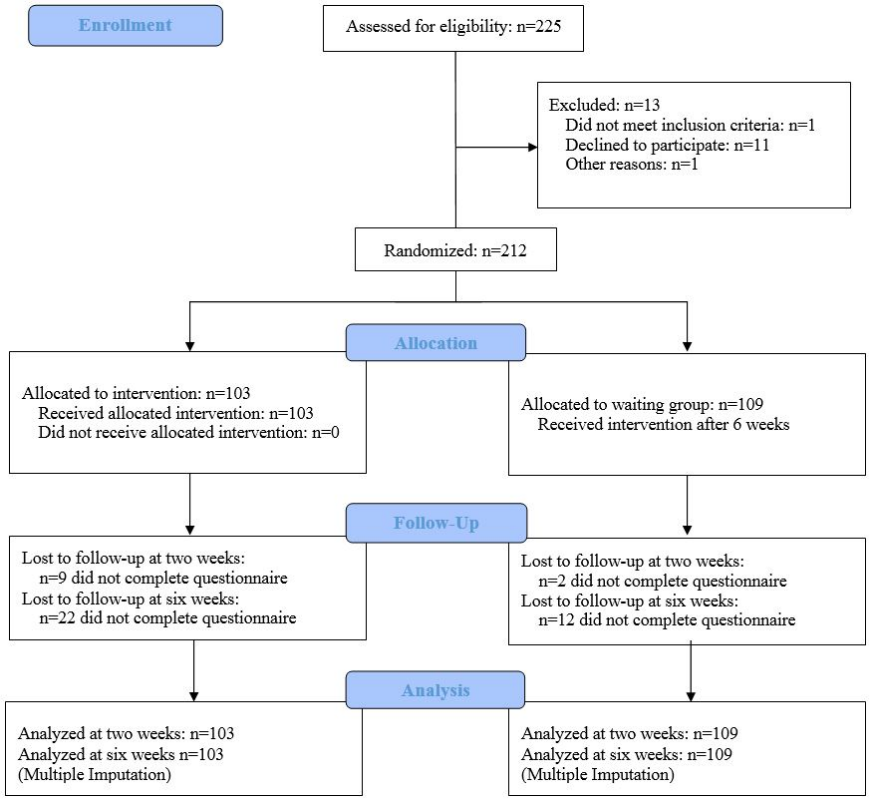
Flowchart of study participation.

The sample consisted of 212 randomized participants who completed the baseline survey. Figure 1 shows the participant flow.

The mean age of participants was 54.1 (SD 11.1) years and 58.8% (124/211) were female. Approximately 73.6% (156/212) of the study sample had read or heard stories and experiences of other patients online before that were unrelated to the intervention provided in the study. Most participants were recruited via the Internet (123/212, 58.0%), 33 (15.6%) were referred to the website by their physician, 25 (11.8%) were recruited via flyers, 8 (3.8%) had been informed by friends, and 23 (10.8%) provided no information about their recruitment path.

**Table 2:** Sociodemographic characteristics of participants in the intervention and control groups at baseline (N=212).

Sociodemographic characteristics		Intervention (n=102-103)	Control (n=107-109)	*P*^a^
**Gender (n (%)**				.17
	Female	55 (53.9)	69 (63,3)	
	Male	47 (46.1)	40 (36.7)	
Age in years, mean (SD)		54.5 (11.8)	53.6 (10.5)	.57
**Family status, n (%)**				.19
	With partner	85 (82.5)	82 (75.2)	
	No partner	18 (17.5)	27 (24.8)	
**Children, n (%)**				.70
	Yes	78 (75.7)	80 (73.4)	
	No	25 (24.3)	29 (26.6)	
**Education (years), n (%)**				.96
	≥13	53 (52.0)	57 (53.3)	
	10	39 (38.2)	39 (36.4)	
	9	10 (9.8)	11 (10.3)	
**Professional training, n (%)**				.06
	No degree	8 (7.8)	1 (0.9)	
	Vocational training	55 (53.9)	53 (49.5)	
	University degree	33 (32.4)	44 (41.1)	
	Other	6 (5.9)	9 (8.4)	
**Employment status, n (%)**				.41
	Employed	30 (29.4)	34 (31.2)	
	Unemployed	4 (3.9)	2 (1.8)	
	Sick leave	22 (21.6)	31 (28.4)	
	Retired	29 (28.4)	32 (29.4)	
	Homemaker	10 (9.8)	4 (3.7)	
	Other	7 (6.9)	6 (5.5)	
**Place of residence (number of inhabitants), n (%)**				.83
	<100,000	71 (68.9)	74 (67.9)	
	100,000-1,000,000	16 (15.5)	20 (18.3)	
	>1,000,000	16 (15.5)	15 (13.8)	

^a^*P* values for group comparisons are based on one-way ANOVAs for age and on chi-square tests for categorical variables.

As shown in Tables 2 and 3, there were no significant differences at baseline between intervention and control participants with respect to sociodemographic or illness and treatment characteristics. Similarly, there were no differences between the groups with regard to having sought health information online before entering the trial, including having read reports of other patients online (Table 4). Groups also did not differ significantly at baseline with respect to the primary and secondary outcomes and depression. However, participants in the control group tended to experience slightly less positive social support than those in the intervention group, with an effect size of eta squared=.02, which would qualify as small [42].

**Table 3:** Illness and treatment characteristics of participants in the intervention and control groups at baseline (N=212).

Illness and treatment characteristics		Intervention, n (%) (n=52-103)	Control, n (%) (n=64-109)	*P*^a^
**Time since diagnosis**				.07
	<2 years before survey	62 (60.2)	61 (56.0)	
	2-3 years before survey	28 (27.2)	42 (38.5)	
	≥4 years before survey	13 (12.6)	6 (5.5)	
**Stoma**				.37
	Yes	25 (24.8)	33 (30.3)	
	No	76 (75.2)	76 (69.7)	
**Metastases**				.92
	Yes	48 (46.6)	48 (44.0)	
	No	52 (50.5)	58 (53.2)	
	Do not know	3 (2.9)	3 (2.8)	
**Relapse**				.72
	Yes	12 (11.7)	11 (10.1)	
	No	91 (88.3)	98 (89.9)	
**Other disease**				.33
	Yes	40 (38.8)	35 (32.4)	
	No	63 (61.2)	73 (67.6)	
**Chemotherapy**				.91
	Completed	53 (58.9)	61 (60.4)	
	Ongoing	29 (32.2)	29 (28.7)	
	Planned or uncertain	5 (5.6)	6 (5.9)	
	Not received	3 (3.3)	5 (5.0)	
**Radiotherapy**				.88
	Completed	33 (63.5)	43 (67.2)	
	Ongoing	2 (3.8)	2 (3.1)	
	Planned or uncertain	2 (3.8)	1 (1.6)	
	Not received	15 (28.8)	18 (28.1)	
**Surgery**				.60
	Completed	88 (89.8)	96 (93.2)	
	Ongoing	1 (1.0)	1 (1.0)	
	Planned or uncertain	8 (8.2)	4 (3.9)	
	Not received	1 (1.0)	2 (1.9)	

^a^*P* values for group comparisons are based on chi-square tests for categorical variables.

**Table 4:** Health information-seeking characteristics and mean scores for patient competence, social support, and depression of participants in the intervention and control groups at baseline (N=212).

Patient characteristics			Intervention	Control	*P*^a^
**Health information-seeking behavior**			n=71-103	n=83-109	
	**Used psycho-oncological support, n (%)**				.59
		Yes	39 (43.8)	35 (39.8)	
		No	50 (56.2)	53 (60.2)	
	**Participates in self-help-groups**				.46
		Yes	12 (16.9)	18 (21.7)	
		No	59 (83.1)	65 (78.3)	
	**Sought Internet health information, n (%)**				.62
		Yes	96 (97.0)	101 (98.1)	
		No	3 (3.0)	2 (1.9)	
	**Had contact with others affected, n (%)**				.81
		Yes	76 (73.8)	82 (75.2)	
		No	27 (26.2)	27 (24.8)	
	**Participated actively in Internet chats and forums, n (%)**				.74
		Yes	19 (23.5)	19 (21.3)	
		No	62 (76.5)	70 (78.7)	
	**Read Internet reports of others affected before entering trial, n (%)**				.87
		Yes	76 (74.5)	80 (75.5)	
		No	26 (25.5)	26 (24.5)	
Self-efficacy for coping with cancer, mean (SD)^b^			99.74 (17.20)	96.27 (19.71)	.20
**Patient competencies, mean (SD)**					
	**Problem-focused**		n=96-103	n=103-108	
		Seeking information	4.00 (0.79)	4.04 (0.78)	.73
		Self-regulation	3.61 (0.51)	3.48 (0.75)	.18
		Patient-physician interaction	4.09 (0.71)	4.00 (0.78)	.40
		Autonomous decision	2.92 (0.82)	2.84 (0.89)	.51
		Interest in social benefits	3.98 (1.42)	3.92 (1.38)	.76
	**Emotion-focused**		n=58-86	n=61-94	
		Coping with distress	3.54 (0.88)	3.32 (0.77)	.14
		Dealing with threat	3.78 (0.56)	3.70 (0.72)	.53
		Low avoidance	3.39 (0.78)	3.35 (0.80)	.74
Depression, mean (SD)^c^			1.58 (1.55)	1.81 (1.51)	.29
**Social support, mean (SD)**			n=103-109	n=102-106	
	Positive support		4.43 (0.62)	4.22 (0.78)	.04
	Distressing interaction		2.00 (0.77)	1.92 (0.70)	.48

^a^*P* values for group comparisons are based on chi-square tests for categorical variables and on one-way ANOVAs for self-efficacy for coping with cancer, patient competence, depression, and social support.

^b^Intervention: n=94; control: n=97.

^c^Intervention: n=101; control: n=105.

### Website Use of Intervention Group

On average, participants in the intervention group visited the intervention website for mean 42.21 (SD 45.64, median 26) minutes in total. The mean number of sessions at the site was 3.43 (SD 2.94, median 3). A mean 40.15 (SD 42.14, median 26) clicks across all sessions suggests that the intervention participants accessed a moderately large number of subpages.

### Primary and Secondary Outcomes at Weeks 2 and 6

Table 5 shows the results of the regression analyses of the scores of the intervention and control group at 2 weeks. As shown by the unstandardized regression weight for the group factor (b group), there were no significant differences at 2 weeks between the intervention and control groups for self-efficacy for coping with cancer as the primary outcome. Furthermore, no significant group differences were determined for the secondary outcome measures of patient competencies, such as self-regulation, coping effectively with emotional distress, dealing explicitly with the threat posed by cancer, and low avoidance. The b coefficients obtained for the respective baseline scores serving as a covariate were generally significant. The corresponding effect sizes (expressed as beta weights) ranged from 0.49 to 0.72, thus indicating large effects [42]. The mean scores tended to be lower at 2 weeks in comparison to baseline (Table 5). Additional group×time repeated measures ANOVAs of changes from baseline to 2 weeks showed a generally significant decrease in all outcome variables except for coping with distress (unimputed data, *F* values not shown, for mean values see Table 5). With values of eta squared less than .06, effect sizes were judged as small [42].

Table 6 presents the results of the regression analyses of the scores of the intervention and control subjects at time 2. Again, intervention and control did not differ with respect to the primary and secondary outcomes as shown by the nonsignificant b coefficients for the group factor. The size of the significant beta weights of the baseline scores entered as covariates ranged from 0.46 to 0.70, also suggesting a large effect of the baseline measure here [42].

**Table 5:** Results of regression analyses of group effects on primary and secondary outcomes at 2 weeks including the respective baseline score as additional predictor.

Outcomes				Participants, mean (SE)^a^			Group effect^a^			Baseline predictor^a^		
				Intervention (n=103)	Control (n=109)	Total (N=212)	b	*P*	beta (range)	b	*P*	beta (range)
**Primary outcome**												
	**Self-efficacy for coping**						–2.25	.21	–0.07, –0.05	0.79	<.001	0.74, 0.76
		Baseline		98.35 (1.66)	94.45 (1.94)	96.34 (1.29)						
		Week 2		96.06 (1.68)	90.71 (2.09)	93.31 (1.36)						
**Secondary outcomes**												
	**Patient competencies**											
		**Self-regulation**					–0.03	.60	–0.03, –0.01	0.83	<.001	0.76, 0.80
			Baseline	3.58 (0.06)	3.48 (0.07)	3.53 (0.05)						
			Week 2	3.49 (0.06)	3.37 (0.08)	3.43 (0.05)						
		**Coping with distress**					–0.13	.07	–0.12, –0.07	0.64	<.001	0.68, 0.72
			Baseline	3.62 (0.08)	3.42 (0.07)	3.52 (0.05)						
			Week 2	3.53 (0.06)	3.27 (0.07)	3.39 (0.05)						
		**Dealing with threat**					–0.05	.52	–0.07, –0.03	.50	<.001	0.49, 0.54
			Baseline	3.74 (0.06)	3.73 (0.07)	3.74 (0.04)						
			Week 2	3.72 (0.06)	3.67 (0.06)	3.69 (0.04)						
		**Low avoidance**					0.03	.68	0.01, 0.04	0.66	<.001	0.66, 0.71
			Baseline	3.40 (0.08)	3.38 (0.08)	3.39 (0.05)						
			Week 2	3.24 (0.07)	3.25 (0.07)	3.25 (0.05)						

^a^Results based on 10 multiple imputations, b coefficient combined (mean), beta coefficients as effect size, minimum and maximum across imputations, group dummy coded with intervention=0, control=1.

**Table 6:** Results of regression analyses of group effects on primary and secondary outcomes at 6 weeks including respective baseline scores as additional predictor.

Outcomes				Participants, mean (SE)^a^			Group effect^a^			Baseline predictor^a^		
				Intervention (n=103)	Control (n=109)	Total (N=212)	b	*P*	beta (range)	b	*P*	beta (range)
**Primary outcome**												
	**Self-efficacy for coping**						–0.00	.99	–0.01, 0.01	0.62	<.001	0.61, 0.64
		Baseline		98.35 (1.66)	94.45 (1.94)	96.34 (1.29)						
		Week 6		93.73 (1.62)	91.29 (2.01)	92.48 (1.30)						
**Secondary outcomes**												
	**Patient competencies**											
		**Self-regulation**					0.09	.23	0.04, 0.08	0.62	<.001	0.63, 0.70
			Baseline	3.58 (0.06)	3.48 (0.07)	3.53 (0.05)						
			Week 6	3.46 (0.06)	3.48 (0.07)	3.47 (0.05)						
		**Coping with distress**					–0.01	.86	–0.03, 0.02	.53	<.001	0.59, 0.65
			Baseline	3.62 (0.08)	3.42 (0.07)	3.52 (0.05)						
			Week 6	3.50 (0.06)	3.38 (0.06)	3.44 (0.04)						
		**Dealing with threat**					–0.02	.75	–0.05, 0.01	0.47	<.001	0.46, 0.54
			Baseline	3.74 (0.06)	3.73 (0.07)	3.74 (0.04)						
			Week 6	3.73 (0.06)	3.70 (0.06)	3.71 (0.04)						
		**Low avoidance**					–0.04	.62	–0.07, 0.01	0.61	<.001	0.61, 0.67
			Baseline	3.40 (0.08)	3.38 (0.08)	3.39 (0.05)						
			Week 6	3.24 (0.08)	3.18 (0.07)	3.21 (0.05)						

^a^Results based on 10 multiple imputations, b coefficient combined (mean), beta coefficients as effect size, minimum and maximum across imputations, group dummy coded with intervention=0, control=1.

## Discussion

### Principal Results

This randomized controlled trial investigated the effects of a website presenting systematically collected and organized patients’ experiences of living with colorectal cancer on self-efficacy for coping with the disease and on patient competencies such as coping with emotional distress or dealing with the life threatening nature of cancer [10]. Participants randomized to the intervention were given access to the website for two consecutive weeks. Contrary to expectations, no intervention effects were found at 2 and 6 weeks after baseline. Also contrary to expectations, primary and secondary outcome scores showed a slight but significant decrease from baseline to follow-up measurements.

In what follows, we will briefly discuss possible explanations for each of these observations. Insofar as these explanations involve factors relating to characteristics of the new website module itself or to its use by participants, they will primarily be discussed in the section comparing these results to prior work. In contrast, explanations that involve methodological factors will be discussed in the limitations section. Considering these factors in more detail may help improve the design of future studies that aim at evaluating websites providing patient narratives on living with (colorectal) cancer. This appears especially important if one shares the conviction that such narratives contain elements that are relevant for empowering cancer patients and helping them develop their coping competencies and coping self-efficacy.

Regarding the slight, but significant, decrease of self-efficacy for coping with cancer and three patient competence scales across time observed in this study, a possible explanation may lie in assuming the operation of a response shift [52]. In the course of the study, participants may have undergone a change in their frames of reference for rating coping self-efficacy and patient competencies. One may speculate, for example, that the observed decrease might reflect some sort of disillusionment resulting from participants’ encountering narratives that describe coping options, which they perceived as beyond their own repertoire of coping behaviors. Then, however, one would expect this change not to occur in the waiting list control group, which is not the case. Thus, this explanation appears rather unlikely. Finally, one could argue that the observed decrease in coping self-efficacy and competence may indicate a regression toward the mean stemming from the self-selection of participants into the trial who already score high on these measures at baseline. Comparing trial participants to the sample of a previous study [10,41] in fact shows them to score significantly higher on the seeking information scale of the competence measure used here. Their scores on the scales used for measuring the secondary outcome criteria are fairly comparable, rendering regression to the mean an implausible explanation. The foregoing discussion focused on possible explanations of an observed decrease in coping self-efficacy and patient competencies. Therefore, we would like to conclude stressing that identifying factors that help patients’ develop their coping self-efficacy and competencies remains an important task for future research.

### Limitations

A major limitation of this study may be that its participants were much younger on average (mean 54.1, SD 11.1 years) than patients with colorectal cancer in Germany in general (mean 71 years) [53]. Although access to and use of the Internet is increasing in all age groups, older patients still appear to be active on the Web to a lesser percentage than younger patients in Germany [54]. Therefore, including participants from this segment of the population of colorectal cancer patients in future research is called for to gain more insight into how websites such as the one studied here may affect these patients. A comparable argument would apply to the potential role of gender in this context, which was beyond the scope of this study. Finally, including patients’ family or friends in such a study might add another facet to future research in this field because these people often support patients in seeking health information on the Internet [55].

Another important limitation may be the fact that far fewer participants could be recruited for the study than suggested by the initial determination of the necessary sample size. This inevitably reduced the power of the trial to detect a treatment effect, if it in fact existed. It would certainly have been preferable to extend the recruitment phase of the study. Unfortunately, this was not possible because of the timeline of the study and the intention to make the newly constructed website available to the public in due time. Nevertheless, achieving the targeted sample size would by no means have guaranteed to establish the hypothesized effect.

### Comparison With Prior Work

Traditional face-to-face psychoeducational interventions in cancer patients have been shown to yield small-to-medium positive effects on distress and quality of life, although problems with study quality and heterogeneity have to be acknowledged [56-59]. Internet-based interventions targeting these domains are gradually appearing and tend to give comparable results [60-62]. This study extended these latter efforts to providing scientifically based narrative information on living with colorectal cancer online and including self-efficacy for coping with cancer and patient competence as outcome criteria in a randomized controlled trial. However, a feature that distinguishes the aforementioned interventions from the website under study is the apparent curricular structure that is typically designed in accordance with the changes desired in the targeted behavioral domain. Also, these interventions appear to require more participant involvement in terms of time investment when progressing through a series of defined tasks for one or more weeks. In contrast, this study allowed participants to explore the site under study according to their immediate personal goals and preferences. As a consequence, they may have utilized the website to a narrower extent than was theoretically possible. The observation that participants in this study spent a limited amount of time using the site is in line with this argument. Therefore, more detailed analyses of patients’ website user behaviors as a mediator of online intervention effects are called for in future research. Beyond this, evaluating the effects of online interventions presenting illness narratives by cancer patients may also profit from supplementary measures of more general psycho-oncological constructs such as the ones used here, with measures capturing subjectively perceived effects and changes more directly. Efforts in that direction might profit from Pols’ research into patients’ knowledge [63,64] that aims at a reconceptualization incorporating patients’ day-to-day coping transactions with illness on a more specific level.

### Conclusions

Regarding self-efficacy for coping with cancer and patient competence, this study found that having access to a new website presenting illness narratives of colorectal cancer patients that have been systematically collected on a scientific basis has no effect compared to a control condition. Possible explanations of this finding may be seen in specific features of the website itself and in features of patients’ on-site usage behavior that might operate as a moderator of online intervention effects on coping self-efficacy and patient competence and other patient-reported outcomes. As a consequence, it may be of importance to analyze patients’ usage behavior in more detail in future research. Furthermore, future research should extend the range of outcome criteria and include measures that more directly reflect patients’ perceived effects of using such a website.

## Abbreviations

CBI: Cancer Behavior Inventory
CONSORT: Consolidated Standards of Reporting Trials
DIPEx: Database of Individual Patients’ Experiences
EORTC: European Organisation for Research and Treatment of Cancer
PHQ: Patient Health Questionnaire
SSUK: Illness-specific Social Support Scale

## Appendix

Multimedia Appendix 1 CONSORT-EHEALTH (v.1.6.1).

## References

[1] Sarrami-Foroushani P, Travaglia J, Debono D, Braithwaite J (2014). Implementing strategies in consumer and community engagement in health care: results of a large-scale, scoping meta-review. BMC Health Serv Res.

[2] Aujoulat I, d'Hoore W, Deccache A (2007). Patient empowerment in theory and practice: polysemy or cacophony?. Patient Educ Couns.

[3] Aujoulat I, Marcolongo R, Bonadiman L, Deccache A (2008). Reconsidering patient empowerment in chronic illness: a critique of models of self-efficacy and bodily control. Soc Sci Med.

[4] Bravo P, Edwards A, Barr PJ, Scholl I, Elwyn G, McAllister M, Cochrane Healthcare Quality Research Group, Cardiff University (2015). Conceptualising patient empowerment: a mixed methods study. BMC Health Serv Res.

[5] Groen WG, Kuijpers W, Oldenburg HS, Wouters MW, Aaronson NK, van Harten WH (2015). Empowerment of cancer survivors through information technology: an integrative review. J Med Internet Res.

[6] Frisch A, Camerini L, Diviani N, Schulz PJ (2012). Defining and measuring health literacy: how can we profit from other literacy domains?. Health Promot Int.

[7] Mancuso JM (2009). Assessment and measurement of health literacy: an integrative review of the literature. Nurs Health Sci.

[8] Rudd R, Schaeffer D, Pelikan JM (2017). Health literacy developments, corrections, emerging themes. Health Literacy Forschungsstand und Perspektiven.

[9] Sykes S, Wills J, Rowlands G, Popple K (2013). Understanding critical health literacy: a concept analysis. BMC Public Health.

[10] Giesler JM, Weis J (2008). Developing a self-rating measure of patient competence in the context of oncology: a multi-center study. Psychooncology.

[11] Heitzmann CA, Merluzzi TV, Jean-Pierre P, Roscoe JA, Kirsh KL, Passik SD (2011). Assessing self-efficacy for coping with cancer: development and psychometric analysis of the brief version of the Cancer Behavior Inventory (CBI-B). Psychooncology.

[12] McCorkle R, Ercolano E, Lazenby M, Schulman-Green D, Schilling LS, Lorig K, Wagner EH (2011). Self-management: enabling and empowering patients living with cancer as a chronic illness. CA Cancer J Clin.

[13] Merluzzi TV, Nairn RC, Hegde K, Martinez SM, Dunn L (2001). Self-efficacy for coping with cancer: revision of the Cancer Behavior Inventory (version 2.0). Psychooncology.

[14] Holman H, Lorig K, Schwarzer R (1992). Perceived self-efficacy in self-management of chronic disease. Self-Efficacy: Thought Control of Action.

[15] Lorig KR, Sobel DS, Stewart AL, Brown BW, Bandura A, Ritter P, Gonzalez VM, Laurent DD, Holman HR (1999). Evidence suggesting that a chronic disease self-management program can improve health status while reducing hospitalization: a randomized trial. Med Care.

[16] Mühlhauser I, Albrecht M, Steckelberg A (2015). Evidence-based health information and risk competence. Ger Med Sci.

[17] Poddar U, Brownlee S, Stacey D, Volk RJ, Williams JW, Elwyn G (2015). Patient decision aids: a case for certification at the national level in the United States. J Clin Ethics.

[18] Pope TM, Hexum M (2013). Legal briefing: Shared decision making and patient decision aids. J Clin Ethics.

[19] Steckelberg A, Berger B, Köpke S, Heesen C, Mühlhauser I (2005). [Criteria for evidence-based patient information]. Z Arztl Fortbild Qualitatssich.

[20] Entwistle VA, France EF, Wyke S, Jepson R, Hunt K, Ziebland S, Thompson A (2011). How information about other people's personal experiences can help with healthcare decision-making: a qualitative study. Patient Educ Couns.

[21] Holmberg C, Waters EA, Whitehouse K, Daly M, McCaskill-Stevens W (2015). My lived experiences are more important than your probabilities: the role of individualized risk estimates for decision making about participation in the Study of Tamoxifen and Raloxifene (STAR). Med Decis Making.

[22] Ziebland S, Herxheimer A (2008). How patients' experiences contribute to decision making: illustrations from DIPEx (personal experiences of health and illness). J Nurs Manag.

[23] Hartzler A, Pratt W (2011). Managing the personal side of health: how patient expertise differs from the expertise of clinicians. J Med Internet Res.

[24] Setoyama Y, Yamazaki Y, Namayama K (2011). Benefits of peer support in online Japanese breast cancer communities: differences between lurkers and posters. J Med Internet Res.

[25] van Uden-Kraan CF, Drossaert CHC, Taal E, Seydel ER, van de Laar MA (2009). Participation in online patient support groups endorses patients' empowerment. Patient Educ Couns.

[26] Khangura S, Bennett C, Stacey D, O'Connor AM (2008). Personal stories in publicly available patient decision aids. Patient Educ Couns.

[27] Bennett KF, von Wagner C, Robb KA (2015). Supplementing factual information with patient narratives in the cancer screening context: a qualitative study of acceptability and preferences. Health Expect.

[28] Pérez M, Sefko JA, Ksiazek D, Golla B, Casey C, Margenthaler JA, Colditz G, Kreuter MW, Jeffe DB (2014). A novel intervention using interactive technology and personal narratives to reduce cancer disparities: African American breast cancer survivor stories. J Cancer Surviv.

[29] Bekker HL, Winterbottom AE, Butow P, Dillard AJ, Feldman-Stewart D, Fowler FJ, Jibaja-Weiss ML, Shaffer VA, Volk RJ (2013). Do personal stories make patient decision aids more effective? A critical review of theory and evidence. BMC Med Inform Decis Mak.

[30] Borzekowski DL, Guan Y, Smith KC, Erby LH, Roter DL (2014). The Angelina effect: immediate reach, grasp, and impact of going public. Genet Med.

[31] Evans DG, Barwell J, Eccles DM, Collins A, Izatt L, Jacobs C, Donaldson A, Brady AF, Cuthbert A, Harrison R, Thomas S, Howell A, Miedzybrodzka Z, Murray A, FH02 Study Group (2014). The Angelina Jolie effect: how high celebrity profile can have a major impact on provision of cancer related services. Breast Cancer Res.

[32] Evans DG, Wisely J, Clancy T, Lalloo F, Wilson M, Johnson R, Duncan J, Barr L, Gandhi A, Howell A (2015). Longer term effects of the Angelina Jolie effect: increased risk-reducing mastectomy rates in BRCA carriers and other high-risk women. Breast Cancer Res.

[33] Haase N, Betsch C, Renkewitz F (2015). Source credibility and the biasing effect of narrative information on the perception of vaccination risks. J Health Commun.

[34] Chirico A, Lucidi F, Merluzzi T, Alivernini F, Laurentiis MD, Botti G, Giordano A (2017). A meta-analytic review of the relationship of cancer coping self-efficacy with distress and quality of life. Oncotarget.

[35] Ziebland S, Herxheimer A, Hurwitz B, Greenhalgh T, Skultans V (2004). The DIPEx Project: collecting personal experiences. Narrative Research in Health and Illness.

[36] Schlesinger M, Grob R, Shaller D, Martino SC, Parker AM, Finucane ML, Cerully JL, Rybowski L (2015). Taking patients' narratives about clinicians from anecdote to science. N Engl J Med.

[37] Engler J, Adami S, Adam Y, Keller B, Repke T, Fügemann H, Lucius-Hoene G, Müller-Nordhorn J, Holmberg C (2016). Using others' experiences. Cancer patients' expectations and navigation of a website providing narratives on prostate, breast and colorectal cancer. Patient Educ Couns.

[38] Newman MA, Ziebland S, Barker KL (2009). Patients' views of a multimedia resource featuring experiences of rheumatoid arthritis: pilot evaluation of www.healthtalkonline.org. Health Informatics J.

[39] Rozmovits L, Ziebland S (2004). What do patients with prostate or breast cancer want from an Internet site? A qualitative study of information needs. Patient Educ Couns.

[40] Albrecht K, Droll H, Giesler JM, Nashan D, Meiss F, Reuter K (2013). Self-efficacy for coping with cancer in melanoma patients: its association with physical fatigue and depression. Psychooncology.

[41] Giesler J, Reuter K, Weis J (2009). Die Veränderung bewältigungsbezogener Selbstwirksamkeitsüberzeugungen im Laufe der onkologischen Rehabilitation - Eine Studie auf der Basis der deutschsprachigen Kurzform des Cancer Behavior Inventory. DRV-Schriften.

[42] Cohen J (2009). Statistical Power Analysis for the Behavioral Sciences.

[43] Giesler J, Weis J (2008). Psychometric properties of the German brief form of the cancer Behavior inventory. Psychooncology.

[44] Kroenke K, Spitzer RL, Williams JB (2003). The Patient Health Questionnaire-2: validity of a two-item depression screener. Med Care.

[45] Mitchell AJ (2012). Clinical utility of screening for clinical depression and bipolar disorder. Curr Opin Psychiatry.

[46] Ullrich A, Mehnert A (2010). Psychometrische Evaluation and Validierung einer 8-Item-Kurzversion der Skalen zur Sozialen Unterstützung bei Krankheit (SSUK) bei Krebspatienten. Klinische Diagnostik und Evaluation.

[47] Ramm G, Hasenbring M (2003). Die deutsche Adaptation der Illness-specific Social Support Scale und ihre teststatistische Überprüfung beim Einsatz an Patienten vor und nach Knochenmarktransplantation. Zeitschrift für Medizinische Psychologie.

[48] Aaronson NK, Ahmedzai S, Bergman B, Bullinger M, Cull A, Duez NJ, Filiberti A, Flechtner H, Fleishman SB, de Haes JC (1993). The European Organization for Research and Treatment of Cancer QLQ-C30: a quality-of-life instrument for use in international clinical trials in oncology. J Natl Cancer Inst.

[49] Arraras JI, Greimel E, Sezer O, Chie W, Bergenmar M, Costantini A, Young T, Vlasic KK, Velikova G (2010). An international validation study of the EORTC QLQ-INFO25 questionnaire: an instrument to assess the information given to cancer patients. Eur J Cancer.

[50] Rubin D (1987). Multiple Imputation for Nonresponse in Surveys.

[51] Little RJ (1988). A test of missing completely at random for multivariate data with missing values. J Am Stat Assoc.

[52] Gerlich C, Schuler M, Jelitte M, Neuderth S, Flentje M, Graefen M, Krüger A, Mehnert A, Faller H (2016). Prostate cancer patients' quality of life assessments across the primary treatment trajectory: 'true' change or response shift?. Acta Oncol.

[53] (2016). Bericht zum Krebsgeschehen in Deutschland 2016.

[54] Czajka S (2010). Statistisches Bundesamt.

[55] Richards R, McNoe B, Iosua E, Reeder A, Egan R, Marsh L, Robertson L, Maclennan B, Dawson A, Quigg R, Petersen A (2016). Cancer information seeking among adult New Zealanders: a national cross-sectional study. J Cancer Educ.

[56] Faller H, Schuler M, Richard M, Heckl U, Weis J, Küffner R (2013). Effects of psycho-oncologic interventions on emotional distress and quality of life in adult patients with cancer: systematic review and meta-analysis. J Clin Oncol.

[57] Germino BB, Mishel MH, Crandell J, Porter L, Blyler D, Jenerette C, Gil KM (2013). Outcomes of an uncertainty management intervention in younger African American and Caucasian breast cancer survivors. Oncol Nurs Forum.

[58] Linden W, Girgis A (2012). Psychological treatment outcomes for cancer patients: what do meta-analyses tell us about distress reduction?. Psychooncology.

[59] Parahoo K, McDonough S, McCaughan E, Noyes J, Semple C, Halstead EJ, Neuberger MM, Dahm P (2013). Psychosocial interventions for men with prostate cancer. Cochrane Database Syst Rev.

[60] Beatty L, Koczwara B, Wade T (2016). Evaluating the efficacy of a self-guided Web-based CBT intervention for reducing cancer-distress: a randomised controlled trial. Support Care Cancer.

[61] Cheung EO, Cohn MA, Dunn LB, Melisko ME, Morgan S, Penedo FJ, Salsman JM, Shumay DM, Moskowitz JT (2016). A randomized pilot trial of a positive affect skill intervention (lessons in linking affect and coping) for women with metastatic breast cancer. Psychooncology.

[62] Classen CC, Chivers ML, Urowitz S, Barbera L, Wiljer D, O'Rinn S, Ferguson SE (2013). Psychosexual distress in women with gynecologic cancer: a feasibility study of an online support group. Psychooncology.

[63] Pols J (2014). Knowing patients-turning patient knowledge into science. Sci Technol Hum Val.

[64] Pols J (2013). The patient 2. many: about diseases that remain and the different forms of knowledge to live with them. Science & Technology Studies.

